# FGF14 modulates resurgent sodium current in mouse cerebellar Purkinje neurons

**DOI:** 10.7554/eLife.04193

**Published:** 2014-09-30

**Authors:** Haidun Yan, Juan L Pablo, Chaojian Wang, Geoffrey S Pitt

**Affiliations:** Department of Medicine, Duke University Medical Center, Durham, United States; Ion Channel Research Unit, Duke University Medical Center, Durham, United States; Department of Neurobiology, Duke University Medical Center, Durham, United States; Vollum Institute, United States

**Keywords:** resurgent sodium current, cerebellum, spinocerebellar ataxia, cerebellar Purkinje neurons, electrophysiology, mouse

## Abstract

Rapid firing of cerebellar Purkinje neurons is facilitated in part by a voltage-gated Na^+^ (Na_V_) ‘resurgent’ current, which allows renewed Na^+^ influx during membrane repolarization. Resurgent current results from unbinding of a blocking particle that competes with normal channel inactivation. The underlying molecular components contributing to resurgent current have not been fully identified. In this study, we show that the Na_V_ channel auxiliary subunit FGF14 ‘b’ isoform, a locus for inherited spinocerebellar ataxias, controls resurgent current and repetitive firing in Purkinje neurons. FGF14 knockdown biased Na_V_ channels towards the inactivated state by decreasing channel availability, diminishing the ‘late’ Na_V_ current, and accelerating channel inactivation rate, thereby reducing resurgent current and repetitive spiking. Critical for these effects was both the alternatively spliced FGF14b N-terminus and direct interaction between FGF14b and the Na_V_ C-terminus. Together, these data suggest that the FGF14b N-terminus is a potent regulator of resurgent Na_V_ current in cerebellar Purkinje neurons.

**DOI:** http://dx.doi.org/10.7554/eLife.04193.001

## eLife digest

The cerebellum is a region of the brain that is involved in motor control, and it contains a special type of nerve cells called Purkinje neurons. Messages travel along neurons as electrical signals carried by sodium ions, which have a positive electric charge. Normally, when a neuron is ‘at rest’, the plasma membrane that surrounds the neuron prevents the sodium ions outside the cell from entering. To send an electrical signal, voltage-sensitive proteins in the membrane called sodium channels open up. This allows the sodium ions to enter the cell by passing through a pore in the channel protein, thereby changing the voltage across the membrane.

Once sodium channels open, they rapidly become ‘locked’ in a closed state, which allows the membrane voltage to return to its original value before another signal can be sent. This locked state also prevents sodium channels from reopening quickly. As a consequence most neurons cannot send successive electrical signals rapidly. Purkinje neurons are unusual because they can send many electrical signals in quick succession—known as rapid firing—without having to be reset each time.

Rapid firing is possible in Purkinje neurons because the channel proteins can be reopened to allow more Na^+^ to enter the cell, but it is not clear how this is controlled. Now, based on experiments on Purkinje neurons isolated from mice, Yan et al. have shown that a protein called FGF14 that binds to the sodium channel proteins can help them to reopen quickly in order to allow rapid firing.

Spinocerebellar ataxia is a degenerative disease caused by damage to the cerebellum that leads to loss of physical coordination. Some patients suffering from this disease carry mutations in the gene that makes the FGF14 protein. Therefore, understanding the role of FGF14 in the rapid firing of Purkinje neurons may aid the development of new treatments for this disease.

**DOI:** http://dx.doi.org/10.7554/eLife.04193.002

## Introduction

Cerebellar Purkinje neurons, which provide the sole output from the cerebellar cortex, display a repetitive firing behavior driven by voltage-gated Na^+^ channels (Na_V_ channels). Fast repetitive firing in Purkinje neurons is promoted by unusual characteristics associated with Na_V_ channel inactivation, whereby channels recover unusually rapidly from inactivation and in doing so pass a ‘resurgent’ sodium current, which helps drive the cell to fire a subsequent action potential. The molecular components underlying the peculiar properties of Na_V_ channels in Purkinje neurons have not been definitively identified.

Several features of Na_V_ currents in cerebellar Purkinje neurons, however, set them apart from Na_V_ currents recorded in other neurons. First, more than half of the Na_V_ current in Purkinje neurons is carried by Na_V_1.6, an isoform for which inactivation is less complete compared to other Na_V_ channels (Raman et al., 1997). Examination of Na_V_ currents in cerebellar Purkinje neurons from *Scn8a*^*−/−*^ mice showed that, compared to the other resident Na_V_ channels, the *Scn8a*-encoded Na_V_1.6 channels have a relatively large ‘late’ (non-inactivating) component and Na_V_1.6 channels have increased availability (depolarized V_1/2_ for steady-state inactivation). Second, Na_V_ channels in cerebellar Purkinje neurons display an unusual transient re-opening during repolarization, which is identified as ‘resurgent’ Na_V_ current (Raman and Bean, 1997). This resurgent current derives from an unblocking of open channels by a peptide moiety that can be eliminated by the intracellular application of trypsin or chymotrypsin proteases into the cytoplasm (Grieco et al., 2005). The blocking particle acts only on open channels and competes with the inactivation process to prevent channels from entering an absorbed, inactivated state. During action potential repolarization, unblocking of these channels then allows Na^+^ influx that can initiate a subsequent action potential (Khaliq et al., 2003). Since the Na_V_ current in Purkinje neurons carried by Na_V_1.6 is relatively resistant to inactivation, it is particularly susceptible to open block by the peptide moiety. Why Na_V_1.6 channels in cerebellar Purkinje neurons are relatively resistant to inactivation, however, is unknown.

Here, we focused on the role of the ion channel regulator FGF14, which is enriched in cerebellar granule and Purkinje neurons (Wang et al., 2002). FGF14 is one of four fibroblast growth factor homologous factors (FHFs), members of the fibroblast growth factor (FGF) superfamily sharing a FGF-like core but having extended N- and C-termini not found in other FGFs. Individual FHFs undergo alternative splicing that generates distinct N-termini, none of which contain a signal sequence (Smallwood et al., 1996; Munoz-Sanjuan et al., 2000). Thus, unlike other FGFs they are not secreted and do not function as growth factors (Smallwood et al., 1996). Instead, FHFs remain intracellular and modulate various ion channels. FHFs can bind directly to the cytoplasmic C-terminal domains (CTDs) of Na_V_ channels (Liu et al., 2001) and regulate Na_V_ channel function (Lou et al., 2005). Further, FGF14 is a potent regulator of both Na_V_1.1 and Na_V_1.6 (Lou et al., 2005; Laezza et al., 2009), the two dominant Na_V_ channels in cerebellar Purkinje neurons (Raman et al., 1997; Kalume et al., 2007). FHFs also regulate voltage-gated Ca^2+^ channels through a mechanism that does not appear to involve a direct interaction (Hennessey et al., 2013; Yan et al., 2013). An ataxia phenotype in *Fgf14*^*−/−*^ mice highlights the role of FGF14 in cerebellar physiology and the development of spinocerebellar ataxia type 27 (SCA27) in patients with either FGF14 haploinsufficiency or a dominant negative *FGF14* mutation (Wang et al., 2002; van Swieten et al., 2003; Dalski et al., 2005) underscores the role of FGF14 in disease.

Using shRNA knockdown of endogenous FGF14 in cultured cerebellar Purkinje neurons and replacement with informative mutants that blocked binding of FGF14 to Na_V_ CTDs and eliminated the FGF14 extended N-terminus, we found that FGF14, and especially the N-terminus of the FGF14b isoform exerts specific kinetic effects on cerebellar Na_V_ channels that support repetitive firing and therefore provides new information on the pathophysiology of SCA27.

## Results

**Figure 1.**
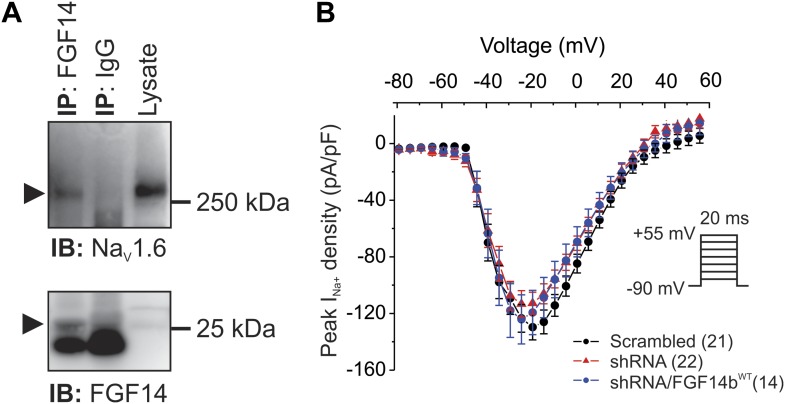
FGF14 is a component of the Na_V_1.6 macromolecular complex in mouse cerebellum, but shRNA knockdown does not affect Na_V_ current density in cultured cerebellar Purkinje neurons. (**A**) Co-immunoprecipitation (IP) of Na_V_1.6 by FGF14 from mouse cerebellum and immunoblot (IB) with the indicated antibodies. M_w_ markers are indicated on right. Arrowheads indicate Na_V_1.6 (top panel) and FGF14 (bottom panel). Intense signal in bottom panel below the FGF14 signal is immunoglobulin light chain. (**B**) Current–voltage relationships (normalized to cell capacitance) of transient Na_V_ currents from cerebellar Purkinje neurons transfected with Scrambled control shRNA (Scrambled), FGF14 shRNA (shRNA), or FGF14 shRNA plus the shRNA-resistant FGF14b^WT^ (shRNA/FGF14^WT^). The number of neurons tested, *N*, is in parentheses. Inset shows schematic of voltage protocol. **DOI:** http://dx.doi.org/10.7554/eLife.04193.003

To test whether FGF14 was a contributor to regulation of Na_V_ current in cerebellar Purkinje neurons, we first confirmed its expression in cerebellum and demonstrated that FGF14 co-immunoprecipitates with Na_V_1.6 channels in mouse cerebellar lysates (Figure 1A). Although FGF14 was previously shown to co-immunoprecipitate with Na_V_1.6 in a heterologous expression system (Laezza et al., 2009), co-immunoprecipitation between FGF14 and Na_V_1.6 channels has not been reported from brain.

To evaluate the consequences of FGF14 haploinsufficiency in SCA27, we used a previously validated shRNA (Yan et al., 2013) to knockdown endogenous FGF14 in cultured cerebellar Purkinje neurons. Knockdown of FGF14 did not affect Na_V_ current density compared to the Na_V_ current density recorded in neurons transfected with a control shRNA (Figure 1B). Because knockout of *Fgf14* reduced spiking activity (Goldfarb et al., 2007) but Na_V_ current density was unaffected in *Fgf14* knockout or knockdown, we hypothesized that the absence of FGF14 must affect Na_V_ kinetic properties in ways that prevent repetitive firing. We therefore examined Na_V_ kinetic properties in more detail.

**Figure 2.**
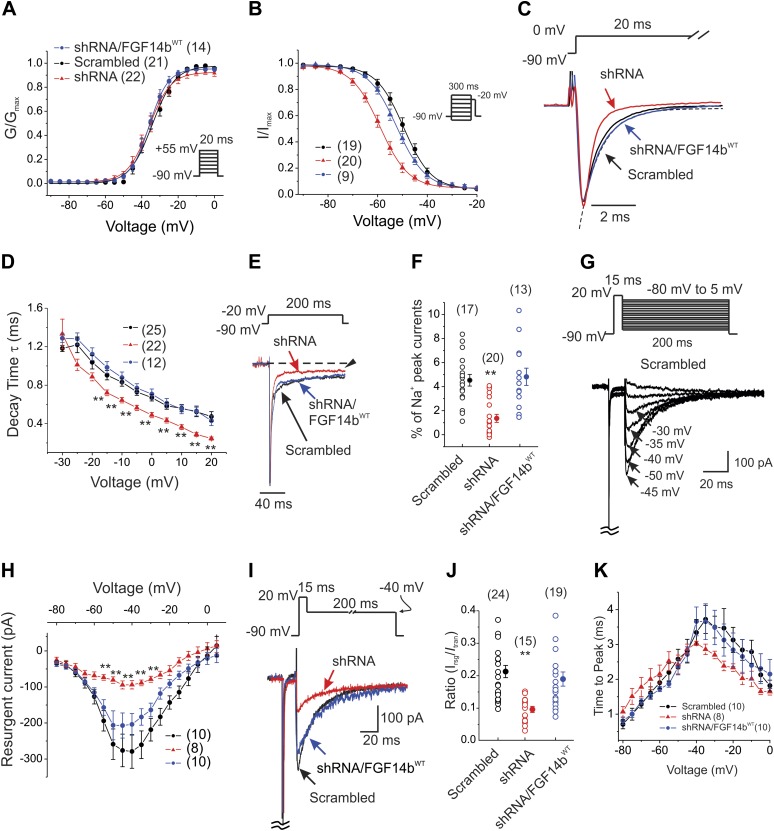
FGF14 knockdown in cerebellar Purkinje neurons affects multiple Na_V_ channel biophysical properties. (**A** and **B**) Voltage-dependence of Na_V_ channel activation and steady-state inactivation in cerebellar Purkinje neurons transfected with Scrambled control shRNA (Scrambled), FGF14 shRNA (shRNA), or FGF14 shRNA plus the shRNA-resistant FGF14b^WT^ (shRNA/FGF14^WT^). The number of neurons tested, *N*, is in parentheses. Inset shows schematic of voltage protocol. (**C**) Exemplar normalized Na_V_ current traces elicited with a step depolarization to 0 mV from a holding potential of −90 mV and an exemplar single exponential fit (for Scrambled) for the time constant (τ) of inactivation (dotted line). (**D**) τ of inactivation at the indicated test voltages. The number of neurons tested, *N*, is in parentheses. **p < 0.01. (**E**) Exemplar normalized TTX-sensitive late Na_V_ currents at −20 mV (measured at 150 ms, arrowhead) from a holding potential of −90 mV. (**F**) Amplitude of late Na_V_ current as a % of peak (transient) Na_V_ current. The number of neurons tested, *N*, is in parentheses. **p < 0.01. (**G**) Voltage-clamp protocol and exemplar TTX-sensitive resurgent Na_V_ currents recorded from cerebellar Purkinje neurons transfected with Scrambled control shRNA. The transient current has been clipped. (**H**) Current–voltage relationship of Na_V_ resurgent currents. The number of neurons tested, *N*, is in parentheses. **p < 0.01. (**I**) Overlay of Na_V_ resurgent currents for the indicated conditions recorded with the indicated voltage protocol. The transient current has been clipped. (**J**) Ratio of peak Na_V_ resurgent current (at +20 mV) to transient Na_V_ current (at −10 mV). The number of neurons tested, *N*, is in parentheses. **p < 0.01. (**K**) Time to peak of Na_V_ resurgent current over a broad range of voltages. The number of neurons tested, *N*, is in parentheses. **p < 0.01. **DOI:** http://dx.doi.org/10.7554/eLife.04193.004

Voltage dependence of activation was unaffected by FGF14 knockdown (Figure 2A), but the V_1/2_ of steady-state inactivation was shifted about −10 mV, thereby decreasing channel availability (Figure 2B and Table 1). When we co-expressed a FGF14b cDNA with synonymous substitutions rendering it resistant to the FGF14 shRNA (FGF14b^WT^), the V_1/2_ of steady-state inactivation was restored to the control voltage; activation was unaffected (Figure 2A–B and Table 1). The ‘rescue’ of steady-state inactivation by expression of the shRNA-insensitive FGF14 provided additional demonstration of the specificity of our shRNA targeting strategy (and see similar rescue for additional parameters below). In addition to the decreased channel availability, we observed that FGF14 knockdown accelerated inactivation kinetics (Figure 2C–D). FGF14 knockdown also reduced the late Na_V_ current (Figure 2E–F). Thus, by several different kinetic measures, FGF14 knockdown in cerebellar Purkinje neurons biased the endogenous Na_V_ channels towards entering the inactivated state. We anticipated, therefore, that FGF14 knockdown should reduce Na_V_ resurgent current amplitude, a key feature that allows cerebellar Purkinje neurons to fire repetitively. Indeed, the amplitude of the resurgent Na_V_ current (Figure 2G–J) was markedly diminished using a well-established protocol to elicit Na_V_ resurgent current (Raman and Bean, 1997). As with the other kinetic properties of Na_V_ currents (above), co-expression of FGF14b^WT^ in the presence of the shRNA provided a complete rescue (Figure 2G–J). While FGF14 knockdown reduced the amplitude of resurgent Na_V_ current, it did not significantly affect the time to peak (Figure 2K).

**Table 1.** Na_V_ current activation and inactivation parameters in cerebellar Purkinje neurons **DOI:** http://dx.doi.org/10.7554/eLife.04193.005

	Activation			Inactivation		
	V_1/2_ (mV)	*K*	n	V_1/2_ (mV)	*K*	n
Scrambled	−34.4 ± 1.5	4.0 ± 0.3	21	−49.7 ± 1.2	4.2 ± 0.1	19
shRNA	−35.0 ± 1.9	4.3 ± 0.5	22	−59.0 ± 1.2**	4.7 ± 0.2	20
shRNA/FGF14b^WT^	−35.5 ± 1.5	3.9 ± 0.3	14	−52.0 ± 0.9	5.2 ± 0.3	9
shRNA/FGF14^RA^	−33.2 ± 1.5	3.5 ± 0.2	12	−50.9 ± 1.4	4.4 ± 0.3	11
FGF14b^WT^	−33.1 ± 1.4	4.5 ± 0.5	14	−50.6 ± 1.3	4.9 ± 0.3	10
FGF14∆^NT^	−33.9 ± 2.0	3.9 ± 0.5	14	−59.1 ± 0.8**	4.7 ± 0.3	15

Mean ± s.e.m. (n), **p < 0.01 compared to Scrambled control.

**Figure 3.**
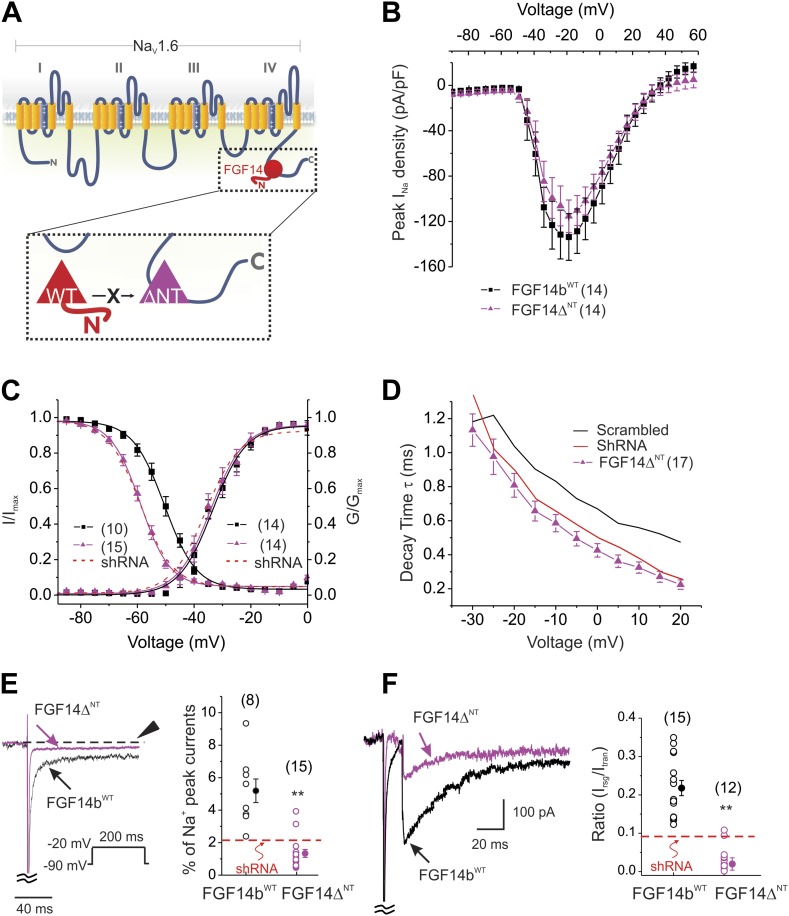
Expression of the dominant negative FGF14Δ^NT^ affects multiple Na_V_ channel biophysical properties indicating essential roles for the FGF14 N-terminus. (**A**) Schematic of endogenous wild type (WT) FGF14 binding to the C-terminus of the Na_V_1.6 α subunit (top) and magnified schematic of the expressed FGF14Δ^NT^ preventing binding of the WT FGF14 (in box). (**B**) Current–voltage relationships (normalized to cell capacitance) of transient Na_V_ currents from cerebellar Purkinje neurons transfected with FGF14b^WT^ or FGF14Δ^NT^. The number of neurons tested, *N*, is in parentheses. The current–voltage relationship for Scrambled control shRNA (from Figure 1) is shown for comparison. (**C**) Voltage dependence of Na_V_ channel activation and steady-state inactivation in cerebellar Purkinje neurons transfected with FGF14b^WT^ or FGF14Δ^NT^. The number of neurons tested, *N*, is in parentheses. The curves for Scrambled control shRNA (from Figure 1) are shown for comparison. (**D**) τ of inactivation at the indicated test voltages. The number of neurons tested, *N*, is in parentheses. (**E**) Exemplar normalized TTX-sensitive late Na_V_ currents at −20 mV (measured at 150 ms, arrowhead) from a holding potential of −90 mV. The number of neurons tested, *N*, is in parentheses. **p < 0.01. (**F**) Overlay of Na_V_ resurgent currents recorded from cerebellar Purkinje neurons transfected with FGF14Δ^NT^ or FGF14b^WT^ and ratio of peak Na_V_ resurgent current (at +20 mV) to transient Na_V_ current (at −10 mV). The number of neurons tested, *N*, is in parentheses. The average for FGF14 shRNA knockdown (Figure 1) is shown for comparison. **p < 0.01. **DOI:** http://dx.doi.org/10.7554/eLife.04193.006

To determine how FGF14 regulates Na_V_ kinetics and Na_V_ resurgent current, we turned our focus to the FGF14 N-terminus. The alternatively spliced N-termini of various FHFs have been shown to exert specific effects on Na_V_ currents (Lou et al., 2005; Rush et al., 2006), including the regulation of inactivation kinetics (Laezza et al., 2009; Dover et al., 2010), but the role of the FGF14 N-termini have not been investigated in cerebellar Purkinje neurons. FGF14b is the predominant FGF14 splice variant expressed in brain (Wang et al., 2000, 2011) and the FGF14 splice variant found in the cytoplasm; FGF14a is localized to the nucleus (Wang et al., 2000). To test the specific roles of the FGF14b N-terminus, we expressed a FGF14 in which the N-terminus was deleted (FGF14Δ^NT^). Because the interaction site for the Na_V_ CTD on FHFs lies within the conserved core domain (Wang et al., 2012), deletion of the N-terminus does not affect interaction with the Na_V_ CTD. Thus, the FGF14Δ^NT^ exerts a dominant negative effect by competing with endogenous FGF14 for interaction with the Na_V_ CTD (Figure 3A). Current density (Figure 3B) and the V_1/2_ of activation (Figure 3C and Table 1) were unaffected. However, expression of FGF14Δ^NT^ shifted the V_1/2_ of steady-state inactivation about −10 mV (Figure 3C), accelerated kinetics of inactivation (Figure 3D), reduced the late Na_V_ current (Figure 3E), and diminished resurgent current (Figure 3F) to a degree similar to or even greater than shRNA knockdown. FGF14Δ^NT^ diminished resurgent Na_V_ current even more effectively than shRNA knockdown of FGF14. As a control, we overexpressed the FGF14b^WT^ that had rescued the parameters affected by shRNA knockdown (Figure 1). Overexpression of FGF14b^WT^ here was without effect (Figure 3 and Table 1). Not only did this serve as a control for FGF14Δ^NT^, but these data suggest that endogenous FGF14b is saturating. These data also suggest that FGF14b's main regulatory component for Purkinje neuron Na_V_ currents lies within the FGF14b N-terminus.

**Figure 4.**
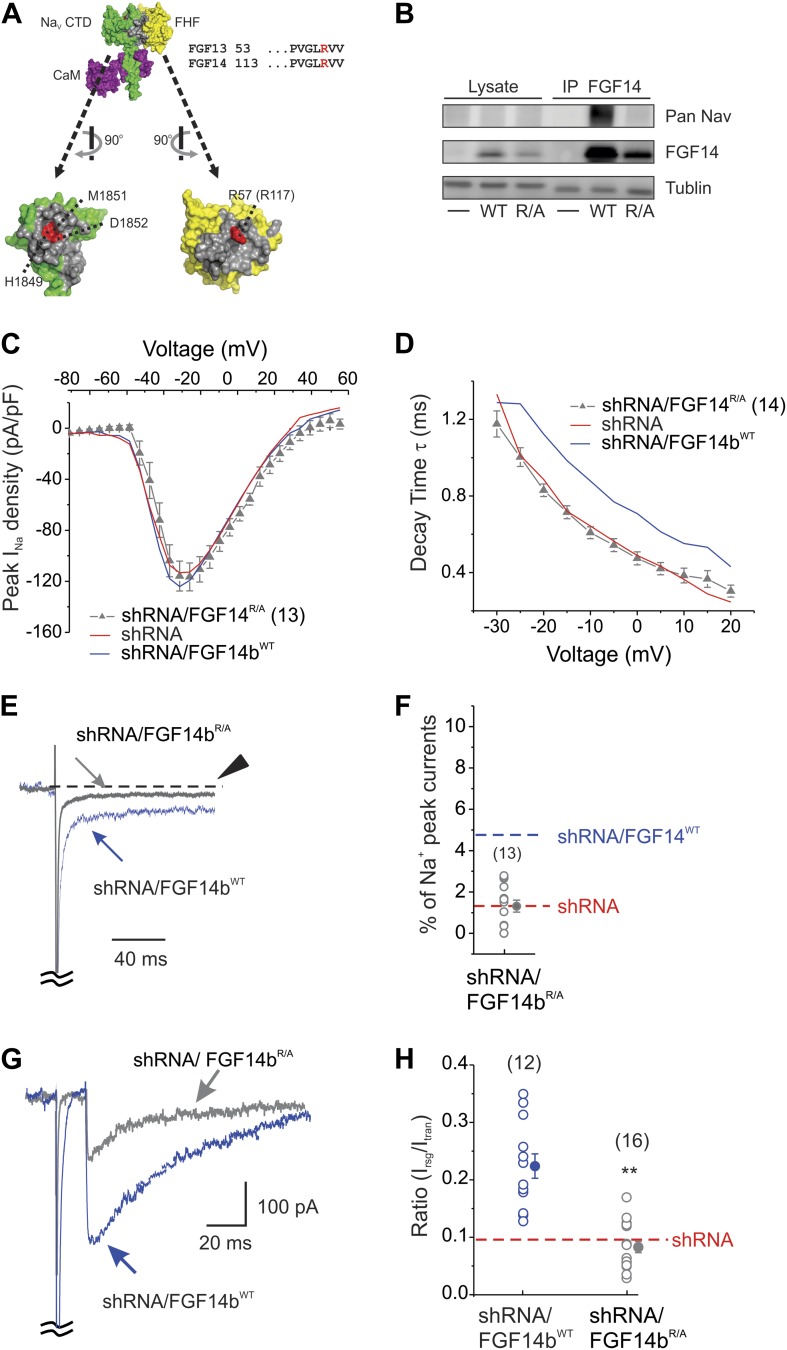
The FGF14b^R/A^ mutant that prevents interaction with the Na_V_ C-terminus cannot rescue Na_V_ kinetic effects of FGF14 knockdown. (**A**) Surface representation of the crystal structure of a ternary complex containing the Na_V_1.5 C-terminus domain (CTD, green), FGF13 (yellow), and calmodulin (purple); the interaction surfaces are colored gray (PDB ID: 4DCK). The critical R57 in FGF13 (equivalent to R117 in FGF14) is indicated in red, as is its binding pocket on the Na_V_1.5 CTD. (**B**) Co-immunoprecipitation (IP) of Na_V_1.6 and FGF14b^WT^ or FGF14b^R/A^ expressed in HEK293 cells, showing that the FGF14b^R/A^ is unable to interact with the intact Na_V_1.6. Immunoblots (IB) were performed with the indicated antibodies. (**C**) Current–voltage relationship (normalized to cell capacitance) of transient Na_V_ currents from cerebellar Purkinje neurons transfected with FGF14b^R/A^. The number of neurons tested, *N*, is in parentheses. The current–voltage relationship for FGF14 knockdown (shRNA) and knockdown rescued with shRNA-insensitive FGF14b^WT^ (shRNA/FGF14b^WT^) from Figure 1 are shown for comparison. (**D**) τ of inactivation at the indicated test voltages. The number of neurons tested, *N*, is in parentheses. (**E**) Exemplar normalized TTX-sensitive late Na_V_ currents at −20 mV (measured at 150 ms, arrowhead) from a holding potential of −90 mV and (**F**) amplitude of late Na_V_ current as a % of peak (transient) Na_V_ current. The number of neurons tested, *N*, is in parentheses. Averages for shRNA and for shRNA/FGF14b^WT^ (see Figure 1) are shown for comparison. (**G**) Overlay of Na_V_ resurgent currents recorded from cerebellar Purkinje neurons transfected with FGF14b^R/A^ or FGF14b^WT^ and (**H**) ratio of peak Na_V_ resurgent current (at +20 mV) to transient Na_V_ current (at −10 mV). The number of neurons tested, *N*, is in parentheses. The average for FGF14 shRNA knockdown (Figure 1) is shown for comparison. **p < 0.01. **DOI:** http://dx.doi.org/10.7554/eLife.04193.007

The dominant negative effect exerted by FGF14Δ^NT^ as it replaced the endogenous FGF14 on the Na_V_ CTD implied that although FGF14 regulates Na_V_ current through its N-terminus, it may require that FGF14 be in direct interaction with the channel. To test this hypothesis specifically, we designed a FGF14b that was unable to bind to the channel CTD and examined whether expression of this mutant could restore the diminished resurgent current resulting from shRNA knockdown of FGF14. Structural determination of a ternary complex containing FGF13, the Na_V_1.5 CTD, and calmodulin had identified key amino acids on the conserved FHF interaction surface that participated in the interaction with the conserved Na_V_ CTD surface (Wang et al., 2012). We focused on a highly conserved Arg in the FHF core domain (Arg57 in FGF13U, equivalent to Arg117 in FGF14b), which inserts into a deep pocket on the Na_V_1.5 CTD binding surface (Figure 4A). We therefore generated the homologous R117A mutant (FGF14b^R/A^) in the FGF14b shRNA-resistant cDNA background and tested whether FGF14b^R/A^ could bind to the intact Na_V_1.6. Although immunoprecipitation of FGF14b^WT^ from lysates of HEK293 cells transfected with FGF14b^WT^ and Na_V_1.6 showed co-immunoprecipitation of Na_V_1.6, the mutant FGF14b^R/A^ was unable to co-immunoprecipitate Na_V_1.6 (Figure 4B). Thus, mutation of R117A in FGF14b prevents interaction with Na_V_1.6. We therefore transfected the shRNA-resistant FGF14b^R/A^ (or the shRNA-resistant FGF14b^WT^) along with the FGF14 shRNA into cultured cerebellar Purkinje neurons to determine the effect of FGF14 interaction with Na_V_1.6 on Na_V_ currents. Expression of FGF14b^R/A^ after shRNA knockdown of endogenous FGF14 had no effect on Na_V_ current density (Figure 4C). In contrast to the rescue afforded by FGF14b^WT^, FGF14b^R/A^ did not rescue the key parameters that lead to increased inactivation observed after FGF14 shRNA. Na_V_ late current and the time constant of inactivation were not different from shRNA (Figure 4D–F). Consistent with these findings, we observed the expected reduction in resurgent Na_V_ current (Figure 4G–H). Thus, the FGF14 N-terminus must exert its key effects through FGF14's direct interaction with the Na_V_ C-terminus.

**Figure 5.**
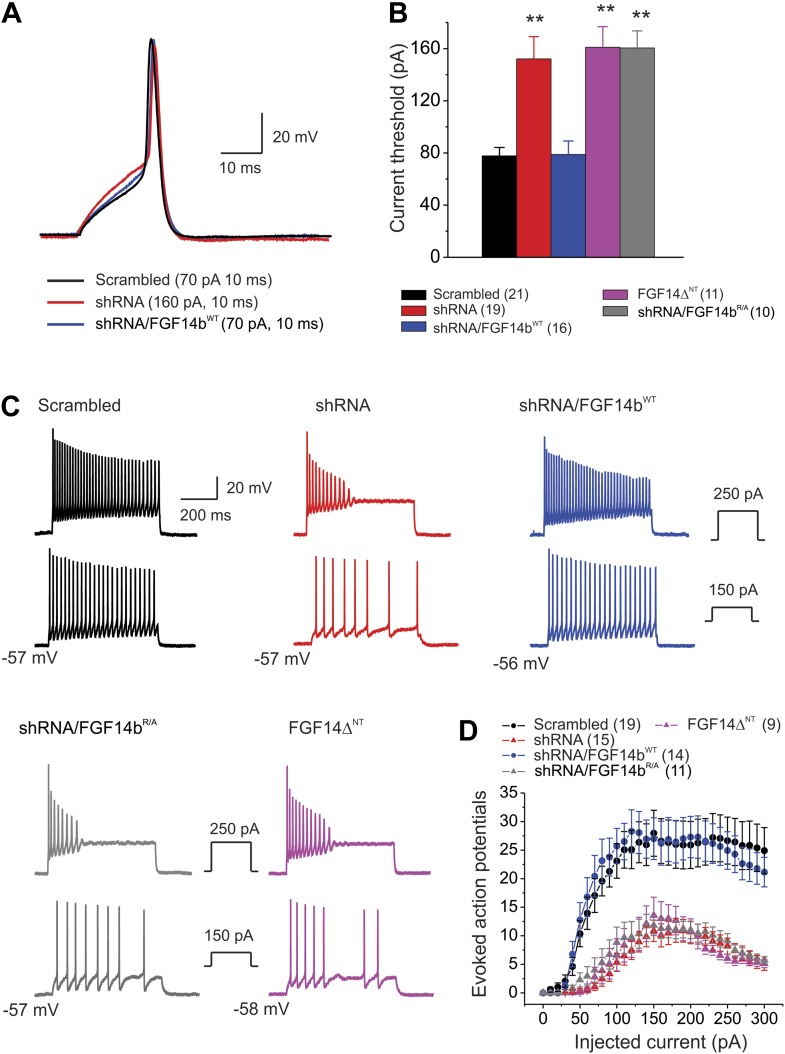
Action potential dynamics and repetitive spiking are dependent upon FGF14 and its N-terminus. (**A**) Overlay of single action potentials evoked with a 10 ms current injection (current amplitude shown in parentheses). (**B**) Current threshold to induce action potentials for the indicated conditions. **p < 0.01. The number of neurons tested, *N*, is in parentheses. Additional summary data are presented in Table 2. (**C**) Example evoked action potentials for the indicated treatments at two separate current injection amplitudes (shown in insets). The resting membrane potential is indicated. (**D**) The number of evoked action potentials for the indicated amplitude of current injection. The number of neurons tested, *N*, is in parentheses. **DOI:** http://dx.doi.org/10.7554/eLife.04193.008

Because the absence of the FGF14 or its N-terminus accelerated Na_V_ channel inactivation, diminished late current, and decreased resurgent current, we anticipated that the resultant bias towards Na_V_ inactivation would adversely affect excitability in cerebellar Purkinje neurons. We tested this hypothesis by determining the effects of the FGF14 and its N-terminus on action potential threshold and on the number of action potentials evoked with depolarizing current injections in current-clamp mode. Under our culture conditions (≤14 days in vitro), we found that 8–10% of cerebellar Purkinje neurons exhibit spontaneous action potentials (firing rate 5–20 Hz) with regular and irregular patterns. When neurons were cultured for extended periods (>20 days in vitro), we observed that ∼50% of Purkinje neurons spontaneously fired—more similar to acutely isolated Purkinje neurons (Raman and Bean, 1999) and cultured Purkinje neurons (Gruol and Franklin, 1987). These data suggest that spontaneous activity depends upon the maturity of the neurons in culture. Because of the difficulty in achieving voltage clamp on Purkinje neurons ≥14 days in vitro, our voltage-clamp experiments were performed on the younger cells (Figures 2–4). Thus, we continued with immature neurons for these experiments, and focused on measuring evoked action potentials rather than spontaneous action potentials. Single action potentials were evoked by a 10-ms current injection with 5-pA increments to determine the current threshold to initiate an action potential. Figure 5A–B and Table 2 show that FGF14 knockdown and expression of the dominant negative FGF14bΔ^NT^ markedly increased the current threshold to produce an action potential; other action potential parameters were not affected. Rescue of FGF14 knockdown by co-expression of FGF14b^WT^ restored the current threshold to control levels. Rescue with the non-interacting FGF14b^R/A^ did not. We also analyzed the number of actions evoked during 600 ms current injection over a wide range of current amplitudes. The number of action potentials evoked was significantly larger in control (scrambled shRNA) neurons than in neurons transfected with FGF14 shRNA. Rescue with FGF14b^WT^, but not FGF14b^R/A^, restored the number of evoked action potentials to control levels. Expression of the dominant negative FGF14bΔ^NT^ reduced the number of evoked action potentials to the level observed after FGF14 knockdown (Figure 5C–D). Thus, as observed for the individual Na_V_ channel kinetic parameters, FGF14b, and its N-terminus specifically, is a critical regulator of neuronal firing and intrinsic membrane properties.

**Table 2.** Intrinsic membrane properties and single action potential (AP) characteristics measured in cerebellar Purkinje neurons **DOI:** http://dx.doi.org/10.7554/eLife.04193.009

	Scrambled	shRNA	shRNA/FGF14b^WT^	shRNA/FGF14b^RA^	FGF14∆^NT^
Input resistance (MΩ)	221.8 ± 15.8 (7)	238.0 ± 49.5 (11)	266.6 ± 24.5 (11)	225.9 ± 10.8 (6)	269.5 ± 30.2 (6)
Resting membrane potential (mV)	−54.5 ± 1.3 (21)	−55.8 ± 1.2 (19)	−54.4 ± 1.0 (16)	−55.4 ± 0.7 (10)	−54.9 ± 1.4 (11)
Current threshold (pA)	77.8 ± 6.4 (21)	142.1 ± 18.5 (19)**	78.8 ± 10.4 (16)	170.5 ± 16.7 (10)**	191.0 ± 22.1 (11)**
AP threshold (mV)	−35.6 ± 1.0 (21)	−32.8 ± 1.2 (19)	−34.5 ± 0.8 (16)	−32.8 ± 1.4 (10)	−32.7 ± 0.8 (11)
AP amplitude (mV)	78.8 ± 3.2 (21)	77.5 ± 2.8 (19)	78.9 ± 4.5 (16)	79.1 ± 4.3 (10)	73.1 ± 4.7 (11)
AP duration (ms)	2.3 ± 0.2 (21)	2.0 ± 0.1 (19)	2.2 ± 0.1 (16)	1.8 ± 0.1 (10)	2.1 ± 0.3 (11)

Mean ± s.e.m. (n), **p < 0.01 compared to Scrambled control.

## Discussion

Although *FGF14* haploinsufficiency is associated with spinocerebellar ataxia (Dalski et al., 2005) and intrinsic excitability of cerebellar Purkinje neurons is reduced in *Fgf14*^*−/−*^ mice (Shakkottai et al., 2009), the detailed multifactorial molecular mechanisms by which FGF14 affects neuronal excitability were not previously known. We had found that FGF14 knockdown in cerebellar granule neurons reduced presynaptic voltage-gated Ca^2+^ currents and thereby affected neurotransmission at the cerebellar granule neuron to Purkinje neuron synapse (Yan et al., 2013). Here, we found that FGF14b in cerebellar Purkinje neurons affects multiple kinetic parameters of Na_V_ channels, thereby reducing the resurgent Na_V_ current that underlies repetitive firing. Together, the accelerated inactivation, reduced channel availability, and decreased late current observed after FGF14 knockdown bias Na_V_ channels to the inactivated state. Thus, the blocking particle that competes with the inactivation gate for open Na_V_ channels is disadvantaged. Consequently, the resurgent current, and the resulting ability to support repetitive firing in Purkinje neurons, is reduced as we observed. Thus, our data show that an essential regulatory feature of FGF14 in cerebellar Purkinje neurons is to slow Na_V_ channel inactivation in order to foster resurgent current.

Resurgent current results from the actions on Na_V_1.6 of a putative blocking particle, which is thought to be the cytoplasmic C-terminal peptide of the Na_V_ auxiliary Na_V_β4 subunit (Grieco et al., 2005). Nevertheless, the actions of Na_V_β4 are not sufficient to generate resurgent Na_V_ current (Chen et al., 2008; Aman et al., 2009), suggesting that other components of the Na_V_ channel complex may be necessary for proper regulation of resurgent Na_V_ current in cerebellar Purkinje neurons. Here, our knockdown experiments and expression of the dominant negative FGF14bΔ^NT^ show that FGF14b, and specifically its N-terminus, strongly influence resurgent Na_V_ current. FGF14 knockdown caused a ∼60% reduction. Expression of FGF14Δ^NT^ was even more efficient, resulting in a ∼85% reduction, perhaps because overexpression of FGF14Δ^NT^ was more efficient in replacing endogenous FGF14b than shRNA knockdown was in eliminating endogenous FGF14b. Thus, FGF14 may cooperate with Na_V_β4 to generate resurgent current, perhaps by biasing Na_V_1.6 channels towards inactivation and thereby disadvantaging the blocking particle. Our results examining effects on total Na_V_ current must be assessed in the context of FGF14 effects not only on Na_V_1.6 channels but also on Na_V_1.1 channel, the other major source of Na_V_ currents in cerebellar Purkinje neurons (Kalume et al., 2007). Those Na_V_ channels are also influence by FGF14 (Lou et al., 2005). Thus, the effects on resurgent current that we attribute to FGF14 may result from FGF14's overall influence of Na_V_ channel kinetics within Purkinje neurons.

Our results may also provide some insight into the more variable phenotypes and decreased penetrance in spinocerebellar ataxia patients with *FGF14* haploinsufficiency (Dalski et al., 2005; Coebergh et al., 2013) vs patients with a *FGF14* mutation (FGF14b^F150S^) found in a large Dutch spinocerebellar ataxia kindred (van Swieten et al., 2003). Studies in hippocampal neurons suggest that the FGF14b^F150S^ mutant exerts a dominant negative mechanism in which the axon initial segment is depleted of Na_V_ channels and Na_V_ current density is reduced (Laezza et al., 2007). Here, we found no effect on Na_V_ current density in cerebellar Purkinje neurons after FGF14 knockdown. A reduction in Na_V_ current density (with the FGF14b^F150S^ mutation) compared to an effect solely on Na_V_ channel kinetics (with FGF14 haploinsufficiency) may explain the higher penetrance and decreased phenotypic variability in patients with the FGF14b^F150S^ mutation.

An important aspect of this study examining effects on Na_V_ channel kinetics was that our analyses were performed in Purkinje neurons. Studying FHFs in their native cellular context appears to be crucial for defining their specific roles. Previous investigations of FGF14-dependent regulation of neuronal Na_V_ channels in heterologous expression systems led to different conclusions from experiments in neurons. For example, expression of Na_V_1.6 and FGF14b^WT^ in ND7/23 cells almost eliminated Na_V_ current density (Laezza et al., 2009) while overexpression of FGF14b^WT^ in hippocampal neurons increased Na_V_ current density (Laezza et al., 2007). Similarly, expression of FGF14Δ^NT^ in ND7/23 cells increased current density, while here we observed that FGF14Δ^NT^ expression in cerebellar Purkinje neurons exerted dominant-negative effects upon Na_V_ channel kinetics, but no consequences for Na_V_ current density. While the etiology of the different results in neurons compared to heterologous expression systems is not known, we speculate that additional regulatory factors present in neurons are necessary for physiologic regulation of Na_V_ channels by FHFs. Indeed, we have observed similarly disparate results for the related FGF13 and the cardiac Na_V_1.5 channel. In HEK293 cells, FGF13 co-expression reduces Na_V_1.5 current density (not shown), but we showed that the role FGF13 in cardiomyocytes is to increase Na_V_ current density (Wang et al., 2011; Hennessey et al., 2013).

Finally, our data also provide information about possible consequences of *FGF14* overexpression, hypothesized as pathologic in rare cases. Some patients with spinocerebellar ataxia, but not controls, harbor synonymous variants in *FGF14*. These variants encode a more frequently used codon than the wild type sequence, leading to the hypothesis that patients with these variants would overexpress *FGF14* as a cause of disease (Dalski et al., 2005). We found, however, that overexpression of FGF14b^WT^ did not affect current density or any of the measured Na_V_ kinetic properties (Figure 3 and Table 1). Thus, if these variants identified in spinocerebellar ataxia patients are truly associated with disease, our data suggest mechanisms other than FGF14 overexpression.

In summary, these data add to a growing appreciation of physiologic and pathophysiologic roles for FHFs in neurons. In the case of spinocerebellar ataxia, our data suggest that FGF14 is a critical regulator of the ability of cerebellar Purkinje neuron Na_V_ channels to support resurgent Na_V_ current and thereby allow Purkinje neurons to fire repetitively. Thus, we provide a molecular understanding for the observation that FGF14 regulates Purkinje neuron intrinsic excitability (Shakkottai et al., 2009). In combination with our data showing that FGF14 also regulates presynaptic voltage-gated Ca^2+^ channels at the cerebellar granule cell to Purkinje cell synapse, these data pinpoint several specific mechanisms by which mutations in *FGF14* underlie spinocerebellar ataxia.

## Materials and methods

### Primary cerebellar neuron culture and transfection

This study was performed in strict accordance with the recommendations in the Guide for the Care and Use of Laboratory Animals of the National Institutes of Health. All of the animals were handled according to approved Institutional Animal Care and Use Committee (IACUC) of Duke University (protocol #A292-13-11). Primary dissociated cerebellar neurons were cultured as previously described (Yan et al., 2013) with some modifications. Briefly, primary cultures were prepared from P6-P8 C57BL6 mice. Cerebella were excised, dissected on ice, digested with 0.25% trypsin for 10 min at 37°C with Dulbecco's Modified Eagle's Medium (DMEM, Sigma, St. Louis, MO), and dissociated into single cells by gentle trituration. The cells were seeded onto coverslips coated with 50 μg/ml poly-D-lysine (Sigma) and 25 μg/ml laminin (Sigma) at a density of 2.5–3.0 × 10^5^ cells/coverslip in DMEM supplemented with 10% heat-inactivated fetal bovine serum (FBS). The neurons were maintained in a humidified incubator in 5% CO_2_ at 37°C. After 15–16 hr, the medium was replaced with Basal Medium Eagle (BME, Sigma) supplemented with 2% B27 (Invitrogen), 5% FBS, 25 μM uridine, 70 μM 5-fluorodeoxyuridine, and 20 mM KCl. Neurons were transiently transfected at 4 days in vitro with 1 μg plasmid DNA and/or shRNA plasmids per coverslip with calcium phosphate. Experiments were performed 8–10 days after transfection (12–14 days in vitro). To achieve good voltage control, all recordings were performed in neurons cultured no longer than 14 days in vitro.

### shRNA and cDNA construction

shRNAs targeted to FGF14 were designed with Invitrogen's RNAi Designer as previously described (Yan et al., 2013) and the sequences were cloned into pLVTHM (Addgene). After evaluating several candidates, we found that the most effective shRNA sequence was 5′ CGCGTGGAGGCAAACCAGTCAACAAGTGCATTCAAGAGATGCACTTGTTGACTGGTTTGC CTCCTTTTTTAT 3′, which was used for the experiments described here. The scrambled shRNA which bears no homology to genes in the rodent genome (Wang et al., 2011) has the sequence: 5′-CGCGTGACCCTTAGTTTATACCTATTCAAGAGATAGGTATAAACTAAGGGTCTTTTTTAT-3′. FGF14 rescue and overexpression experiments were performed with FGF14 constructs (either full-length or with the N-terminus truncated) cloned into pcDNA3.1 also containing tdTomato. Mutagenesis to obtain FGF14b^R/A^ was performed using QuikChange (Agilent). All plasmids were verified by sequencing.

### Electrophysiological recordings

Purkinje neurons were identified by their characteristic teardrop morphology and large size. Whole-cell Na_V_ currents and membrane voltage were recorded at 23–25°C using an EPC 10 USB patch amplifier (HEKA Elektronik). The signal was filtered at 2.9 Hz and digitized at 20 Hz. Na_V_ currents were recorded with an extracellular solution containing (in mM) 124 NaCl, 20 TEA-Cl (tetraethylammonium Chloride), 5 HEPES (4-(2-hydroxyethyl)-1-piperazineethanesulfonic acid), 10 glucose, 0.3 CdCl_2_, 2 BaCl_2_, and 4-AP (4-aminopyridine). NaOH was added to achieve pH 7.3 (300–310 mOsm). Borosilicate glass patch pipettes (resistances within 3–4 MΩ) were filled with the following internal solution (in mM): 125 CsF, 10 NaCl, 10 HEPES, 15 TEA-Cl, 1.1 ethylene glycol tetraacetic acid (EGTA), and 0.5 Na-guanosine-5′-triphosphate (Na-GTP), pH adjusted to 7.3 with CsOH (290–300 mOsm). Tetrodotoxin (TTX)-sensitive Na_V_ currents were isolated by subtraction of recordings performed in 1 μM TTX from the control recordings. Series resistance was compensated >70%. Current-clamp recordings were performed after obtaining seal resistances >1.2 GΩ. The internal solution was (in mM) 130 K-gluconate, 5 NaCl, 2 MgCl_2_, 10 HEPES, 4 Mg-ATP, 0.5 Na-GTP, 0.2 EGTA, and 10 phosphocreatine. KOH was used to obtain pH 7.3 (290–300 mOsm). The external solution was (in mM) 140 NaCl, 5 KCl, 2 CaCl_2_, 1 MgCl_2_, 5 HEPES, and 10 glucose, pH adjusted to 7.3 with NaOH. Resting membrane potential was directly measured in current-clamp mode after membrane rupture and only cells with a resting membrane potential more negative than −50 mV were studied. The liquid junction potential was not corrected. Input resistance was determined from membrane voltage deflection, evoked by 600-ms hyperpolarizing current injections (0 to −300 pA in steps of 50 pA) and calculated from the measured slope. Single action potentials were elicited by 10 ms depolarizing current injections with 5-pA increments. All drugs were from Sigma Aldrich, except for TTX (Abcam Biochemicals).

### Protocols and data analysis

Data analysis was performed using FitMaster (HEKA), Excel (Microsoft), and Origin software. All averaged data are presented as mean ± SEM. Statistical significance was determined using Student's *t* or one-way ANOVA tests. Calculated p values of ≤0.05 were accepted as evidence of statistically significant differences. For current amplitude, neurons were held at −90 mV and transient Na_V_ current was elicited by depolarizing pulses of 20 ms from −90 mV to +55 mV in 5-mV increments. Current density was obtained by normalizing peak Na_V_ current to membrane capacitance. Resurgent Na_V_ current was evoked with repolarizing steps from +20 mV to a range of voltages between −80 mV and +5 mV in 5-mV increments for a 200-ms test pulse after 15 ms conditioning steps. Na_V_ activation curves were obtained by transforming current data to conductance (G), with the equation G = *I*_Na_/(E_m_ − E_rev_), where: *I*_Na_ is the peak current; E_m_ is the membrane potential; and E_rev_ is the reversal potential of *I*_Na_; and fitted with a Boltzmann equation of the form: G = G_max_/[1 + exp(*V*_*1/2*_ *− V*)/*k*], where G_max_ is the extrapolated maximum Na^+^ conductance, V is the test voltage, V_1/2_ is the half-activation voltage, and *k* is the slope factor. For Na_V_ steady-state inactivation, a voltage step to −20 mV for 20 ms was applied from a holding potential of −90 mV to preferentially activate *I*_Na_ after pre-pulse conditioning voltage steps of 300 ms in duration (ranging from −90 to +20 mV) in 5-mV increments. Steady-state inactivation curves were constructed by plotting the normalized peak current amplitude elicited during the test pulse as a function of the conditioning pre-pulse. A Boltzmann relationship, *I*/*I*_max_ = (1 + exp((*V* − *V*_1/2_)/*k*))^−1^ was used to fit the data where *I*_max_ is current elicited by the test pulse after a −90-mV prepulse; *V*_1/2_ is half-inactivation voltage; *k* is the slope. Late Na_V_ current was measured at 150 ms during a 200 ms depolarizing pulse to −20 mV. The rate of decay of the transient Na_V_ current (τ) was obtained from a single exponential function, *I*_*(t)*_ = *I*_*Na*_ exp ^(−t/τ)^+ *I*_*SS*_, where *I*_*(t)*_ is the amplitude of the current at time *t*; *I*_*SS*_ is the steady-state current during a single voltage step.

In current clamp, action potential amplitude was measured as the peak voltage with respect to the baseline 10 ms before the peak of the action potential. The action potential duration was evaluated at half-amplitude. Evoked action potentials were elicited by injecting a depolarizing current from 0 pA to 300 pA for 600 ms duration with 10-pA increments.

### Immunoprecipitation and Western blotting

Cerebella were isolated from two 5-week-old C57BL/6J mice and homogenized with a mortar and pestle in 8 ml of lysis buffer composed of 150 mM NaCl, 50 mM Tris-Cl, 1% Triton, and 1% sodium deoxycholate. Lysate was incubated on ice for 2 hr, passed through a 20g needle 20 times, and rocked end over end for 30 min at 4°C. The lysate was cleared of insoluble material by centrifugation at 4000 rcf at 4°C for 30 min followed by a second centrifugation at 17,100 rcf at 4°C for 10 min. The lysate was preincubated with 30 μl of Protein A/G (Santa Cruz Biotechnology) beads for 2 hr at 4°C. The beads were gently spun down at 0.4 rcf for 1 min to separate them from the lysate, which was then incubated with 20 μg of either anti-FGF14 (NeuroMab) or non-immune mouse IgG (Santa Cruz Biotechnology) overnight at 4°C. In order to immunoprecipitate protein complexes, 30 μl of Protein A/G beads were added and incubated with the lysate for 2 hr. Beads were then washed three times with lysis buffer followed by elution in 2× LDS Sample Buffer (Novex NuPage) plus 10 mM dithiothreiotol. Protein samples were run on 8–16% Tris-glycine SDS page gels and transferred to PVDF membranes. Primary antibodies used were rabbit polyclonal anti Na_v_1.6 from Millipore (1:200), anti-β-tubulin from Sigma (1:1000) as a loading control, and rabbit polyclonal anti-FGF14 (1:200), which was a kind gift from the Ornitz lab (Washington University, St. Louis). HEK293T cells on 100-mm plates were transfected with Lipofectamine 2000 (Invitrogen) when cells were at ∼60% confluency with 9 μg of Na_V_1.6 and 3 μg of FGF14b^WT^ or FGF14b^R/A^. Cells were washed with ice-cold PBS 60 hr after transfection, and cell lysates were prepared with the addition of lysis buffer containing 150 mm NaCl, 50 mm Tris–HCl, pH 7.5, 1% Triton with protease inhibitor (Roche). The pelleted cells were pipetted up and down 20 times with lysis buffer, incubated at 4°C for 1 hr and then centrifuged at 16,000×*g* for 10 min at 4°C. Immunoprecipitation was performed on 2 mg of lysate with 2 μg of anti-FGF14 antibody (Neuromab). The samples were rocked gently at 4°C for 1 hr followed by addition of 30 μl of protein A/G-agarose slurry. The samples were rotated overnight at 4°C and microcentrifuged at 7000 rpm for 2 min. After washing with lysis buffer three times, 40 μl of loading buffer was added to the pellet, and protein was eluted from the beads by heating at 70°C for 20 min. The samples were subjected to NuPAGE 8–16% Bis-Tris gels (Invitrogen). The proteins were transferred to nitrocellulose membranes and subsequently immunoblotted with the anti-FGF14, anti-pan Na_V_ antibody, and anti-tubulin antibody.

## Additional files

### Major dataset

The following previously published dataset was used:

Wang C, Chung BC, Yan H, Lee SY, Pitt GS, 2012, Crystal structure of the C-terminus of voltage-gated sodium channel in complex with FGF13 and CaM, http://www.pdb.org/pdb/explore/explore.do?structureId=4DCK, Publicly available at RCSB Protein Data Bank.
